# Perceptions about data-informed decisions: an assessment of information-use in high HIV-prevalence settings in South Africa

**DOI:** 10.1186/s12913-017-2641-1

**Published:** 2017-12-04

**Authors:** Edward Nicol, Debbie Bradshaw, Jeannine Uwimana-Nicol, Lilian Dudley

**Affiliations:** 1 Burden of Disease Research Unit, South African Medical Research Council, Cape Town, South Africa; 2 Division of Health Systems and Public Health, Faculty of Medicine and Health Sciences, Stellenbosch University, Cape Town, South Africa; 3 School of Public Health and Family Medicine, University of Cape Town, Cape Town, South Africa; 4 School of Public Health, University of the Western Cape, Bellville, South Africa; 5 School of Public Health, University of Rwanda, Kigali, Rwanda

**Keywords:** Data-use, Use of information, Data accuracy, Promotion of information-use, Culture of information-use, PMTCT, Barriers to information use, Competence

## Abstract

**Background:**

Information-use is an integral component of a routine health information system and essential to influence policy-making, program actions and research. Despite an increased amount of routine data collected, planning and resource-allocation decisions made by health managers for managing HIV programs are often not based on data. This study investigated the use of information, and barriers to using routine data for monitoring the prevention of mother-to-child transmission of HIV (PMTCT) programs in two high HIV-prevalence districts in South Africa.

**Methods:**

We undertook an observational study using a multi-method approach, including an inventory of facility records and reports. The performance of routine information systems management (PRISM) diagnostic ‘Use of Information’ tool was used to assess the PMTCT information system for evidence of data use in 57 health facilities in two districts. Twenty-two in-depth interviews were conducted with key informants to investigate barriers to information use in decision-making. Participants were purposively selected based on their positions and experience with either producing PMTCT data and/or using data for management purposes. We computed descriptive statistics and used a general inductive approach to analyze the qualitative data.

**Results:**

Despite the availability of mechanisms and processes to facilitate information-use in about two-thirds of the facilities, evidence of information-use (i.e., indication of some form of information-use in available RHIS reports) was demonstrated in 53% of the facilities. Information was inadequately used at district and facility levels to inform decisions and planning, but was selectively used for reporting and monitoring program outputs at the provincial level. The inadequate use of information stemmed from organizational issues such as the lack of a culture of information-use, lack of trust in the data, and the inability of program and facility managers to analyze, interpret and use information.

**Conclusions:**

Managers’ inability to use information implied that decisions for program implementation and improving service delivery were not always based on data. This lack of data use could influence the delivery of health care services negatively. Facility and program managers should be provided with opportunities for capacity development as well as practice-based, in-service training, and be supported to use information for planning, management and decision-making.

## Background

The use of information to influence policy, program action and research has been described as a vital output of a functional health information system [1–3]; however, for information to be used, data must first be processed and analyzed into a usable format [4]. More emphasis has been placed on data collection in low- and middle-income countries (LMICs) than on information-use. Limited information-use is in part related to the suboptimal quality of data generated by the routine health information systems (RHIS) [5–11], and the absence of a culture of information-use [12–14] defined as “*the capacity and control to promote values and beliefs among members of an organization by collecting, analyzing and using information to accomplish the organization’s goals and mission”* [3].

Substantial investments to promote and improve data-use have been made by organizations such as MEASURE Evaluation [15]. Tools and interventions have also specifically been developed to encourage data demand and use in LMICs. For instance, Nutley and Reynolds created a logic model for strengthening the use of health data in decision making [4]. Based on work by Aqil et al. [3], the Health Metric Network [1, 16], and Patton [17], this model involves the use of eight interventions that aim to improve data demand and use: assessing and improving the data-use context; engaging data users and data producers; improving data quality; improving data availability; identifying information needs; building capacity in data-use core competencies; strengthening organizations’ data demand and use infrastructures; and monitoring, evaluating, and communicating results of data-use interventions [4]. This model has been applied in Ivory Coast to improve the use of data in decision-making, which increased information-use at the district level from 40% in 2008 to 70% in 2012, through building linkages between data collection and decision-making processes such as program review, planning, advocacy and policy development [18].

Despite increasing demand for and the availability of data, which are often not accurate and reliable [6, 7, 10, 19–22], many decisions made by health managers in terms of planning and resource allocation for managing HIV programs are still not based on data [13, 23, 24]. The inadequate use of information generated from RHIS is alarming, considering the investments made and resources channeled towards collecting such data. To highlight the magnitude of this problem, 120 countries globally were asked to self-rate their performance in terms of information-use in HIV program planning, management, and implementation, using a five-point Likert scale from 0 (low) to 5 (high). More than half of the countries surveyed by the United Nations’ General Assembly (UNGASS)’ National Composite Policy Index data focusing on addressing monitoring and evaluation (M&E) systems, rated their data-use experience as below average. Only 48% of the 42 countries in sub-Saharan Africa, including South Africa, rated their data-use experience in HIV planning and implementation as above average [25].

In South Africa, almost 16 years after the implementation of the district health information system (DHIS), the major source of primary health care (PHC) information, data from the system have not been adequately utilized to effectively inform policies and service delivery [26], leaving data producers with the perception that data collection is only for reporting purposes. Reviews of the prevention of mother-to-child transmission of HIV (PMTCT) program have highlighted challenges such as the lack of data to monitor progress across the PMTCT cascade [21, 27]. In the absence of accurate and reliable data, it is difficult, if not impossible, to make evidence-based resource-allocation decisions to increase the efficiency and effectiveness of the PMTCT program. The non-use of information for planning may pose a considerable challenge for policy makers in terms of decisions about investment in HIV prevention and treatment [28]. It is therefore important to use data to identify whether the interventions are effective in curtailing new HIV transmission, as well as averting new deaths in children and their mothers.

The insufficient use of data for HIV prevention has an impact on the supply of treatment, illustrated by a recent national crisis of antiretroviral (ARV) stock-outs in South Africa, which threatened the lives of thousands of patients and undermined efforts to fight HIV [29]. This crisis could have been averted with proper planning and budgeting informed by data from PHC facilities. (Pillay Y, Rohde J, Van den Bergh C: The District Health Information System: a briefing document for Department of Health managers, unpublished) suggest that, despite the routine collection of data in all South African public health facilities, very little of the information is used by decision-makers. The literature highlights several reasons for the insufficient use of information for planning and management purposes. These include the lack of a reliable data source [30]; lack of skills for data interpretation and utilization [31]; problems relating to the data-collection processes such as poor-quality data; and, incomplete and untimely data [32].

There is paucity of data in South Africa about barriers to using information for management purposes. This study sought to investigate the use of information, and barriers to using routine health information for program monitoring in the context of maternal and child health programs in two high HIV-prevalence districts in South Africa.

## Methods

### Study design, setting and participants

We undertook an observational study using a multi-method approach, including an inventory of registers, monthly reports and meeting minutes at each of the 57 selected health facilities in two districts with high HIV prevalence, using the performance of routine information systems management (PRISM) diagnostic ‘Use of Information’ tool [2, 3]. The facilities selected were 27 heath facilities in the Amajuba District (KwaZulu-Natal Province) and 30 in Khayelitsha and the Eastern Sub-districts in the Cape Metro/City of Cape Town District (Western Cape Province). The study setting and sampling have been extensively described elsewhere [7, 33]. We conducted in-depth interviews with 22 purposively selected health-service personnel between July and December 2013 to explore their experiences regarding PMTCT data production and use, and their perceptions about information-use barriers. Participants were selected based on their positions and experience with either producing PMTCT data and/or using data for management purposes. The key informants included district managers/coordinators, sub-district coordinators, PMTCT program managers, M&E officers, facility managers (FMs), and staff involved in data collection (Table 1).

**Table 1:** Participants interviewed by organizational level

Function	Facility Level		Sub-district Level		District Level		Province Level		# of staff interviewed
	KZN	WC	KZN	WC	KZN	WC	KZN	WC	
PIO							1	2	3
PMTCT/HAST (M&E)	1			2	1			1	5
DIO					1	2			3
DM					1				1
SDM				2					2
HIO				1					1
FM	3	2							5
FIO	1								1
CEO	1								1
Total	6	2		5	3	2	1	3	22

*PIO* Provincial information officer, *PMTCT* Prevention of mother-to-child transmission of HIV, *HAST* HIV/AIDS, STI & TB coordinators, *M&E* Monitoring and evaluation, *DIO* District information officer, *DM* District manager, *SDM* Sub-district manager, *HIO* Health information officer, *FM* Facility managers, *FIO* Facility information officer, *CEO* Chief executive officer (Hospital), *KZN* KwaZulu-Natal, *WC* Western Cape

### Data collection and analysis

#### Quantitative: Promotion and use of information

Two teams of two trained fieldworkers each reviewed data from 228 reports in all 57 facilities for evidence of data-use measured using two criteria: (1) the availability of reports such as feedback, quarterly reports, health services, minutes of meetings; and, (2) reviews of these reports for evidence of information-use. A set of weighted elements used to calculate this indicator include: RHIS report production; frequency of RHIS reports; types of reports produced; display of information at the facility level; use of information in available reports at facility; types of decisions based on types of analyses; discussion and decisions based on RHIS information; and, promotion and supervision by the district office. Furthermore, the promotion of information-use at the facility level indicates whether mechanisms and processes that encourage information-use are in place. These were captured and analyzed using simple descriptive statistics, such as measures of frequency and measures of central tendency.

#### Qualitative procedures

The principal investigator and a research assistant who had completed training in qualitative data-collection processes conducted the individual interviews in English, each session lasting 20–45 min. An interview schedule was developed with a list of predetermined sets of questions and themes on data quality, information-use, training, skills and information flow. This schedule served as a checklist to ensure the same questions were administered to all key informants in order to elicit systematic responses that could be easily categorized and analyzed. A trained researcher transcribed the digital audio recordings from the interviews. The transcripts were proofread and repeatedly read, first by the research assistant, and later by the lead author, who compared the transcripts to the original audio recordings. Errors were then corrected.

#### Data analysis

We analyzed the qualitative data using a general inductive approach based on the techniques of systematically identifying emerging themes, categories, or patterns from the data [34]. The lead author and an experienced qualitative analyst independently coded responses from all participants by examining the transcripts, and identifying similar emerging themes. These independent analyses were then compared for consistency and discrepancies were identified through critical evaluation of the sets of themes. The source quotes were reviewed and agreed upon, and a final thematic report was generated from the combined analyses. Results were then shared with program managers and staff at the district level for validation and the findings were revised based on feedback from the managers. Direct quotations are used to illustrate key issues and themes.

#### Observations

The interviews were complemented by observations at facility level, which included observing the availability of any form of reports such as wall charts, quarterly reports, feedback reports or health service reports. Field workers were required to observe the display of maternal and child health-related information, in the form of graphs, charts or tables at each study facility, and to verify if the displayed information was updated. These were collated and compared across facilities.

## Results

### Information-use

**Fig. 1:**
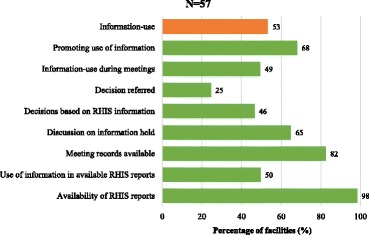
Percentage distribution of information-use at the facility

The percentage distribution of information-use at the facility during the 3 months prior to the study is presented in Fig. 1 and includes a district-level indicator, ‘*Promoting use of information’*, to compare against ‘*Information-use’*. The study shows that whereas about two-thirds of the facilities reported having mechanisms and processes to facilitate information-use (*‘Promoting use of information’*), evidence of information-use (i.e., indication of some form of information-use in available RHIS reports) was demonstrated in 53% of the facilities.

Despite the availability of RHIS reports in 98% of the facilities, only half used the information in these reports. While 82% of the facilities claimed they kept official records of meetings held during the 3 months preceding the survey, only about two-thirds discussed RHIS findings such as patient utilization, disease data, service coverage or medicine stock-outs in meetings.

**Fig. 2:**
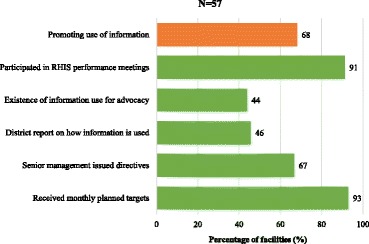
Distribution of promotion and use of information from the routine health information system

An overview of elements assessed for the promotion of information-use is presented in Fig. 2. In over 90% of the facilities surveyed, managers reported having received annual/monthly planned targets from the district offices that were based on RHIS information, and that they had participated in meetings at the district level to discuss RHIS performance over the 3 months prior to the survey. However, managers reported that documentation showing information-use for advocacy purposes, and examples from the district offices of how RHIS information had been successfully utilized in the past, were not available in more than half of the surveyed facilities.

**Fig. 3:**
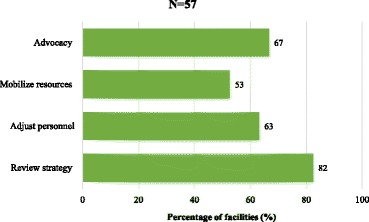
Information-use for decisions in available reports at the facility

Figure 3 shows the distribution of information-use for specific decisions in available reports at the facility. Despite 82% of the facilities having reported the use of RHIS information to review strategies for achieving performance targets, only about half actually used the information to make decisions on resource mobilization based on a comparison of services. However, two-thirds of the facilities reported having used the information to advocate for additional resources.

**Fig. 4:**
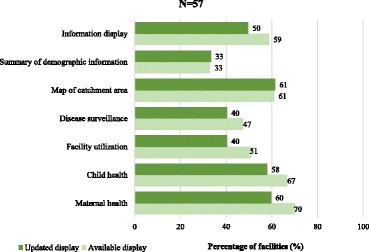
Proportion of facilities displaying updated information from the routine health information system

The survey also assessed the display of RHIS-related information that had been updated at least 6 months prior to the survey. Facilities were observed for any type of information on maternal and child health services, including information on facility utilization, population by target groups, and the presence of a map of the facility catchment area. About 60% of the facilities displayed RHIS information, but only in half of the facilities was the displayed information updated (Fig. 4). Information on maternal and child health was displayed in more than two-thirds of the facilities, while about 60, 47, and 50% of the facilities had maps of their catchment areas, displayed information on disease surveillance, and information on facility utilization, respectively.

### Barriers to information-use

The perceptions of health information personnel about barriers to information-use for planning purposes were explored using a qualitative approach. The guiding questions used to elicit information during data collection were informed by the Information Cycle Model [35] and the Health Metrics Network Framework [1]. Several themes and categories relating to information-use, and reasons why information is insufficiently used for monitoring purposes were identified. Table 2 presents a summary of the general themes and categories about data collection and use of information relating to the experiences of staff at different levels of the health information systems. Four themes (use of information, barriers to information-use, data-quality issues, and reasons for poor data collection), four sub-themes (human resources, equipment, validation issues, and training), and 39 categories relating to information-use and barriers to using information for monitoring purposes emerged from the data. Some of the themes are similar across the cadres of health professionals and have been categorized accordingly. We present in detail with illustrative quotes some of the themes observed across the different cadres interviewed.

**Table 2:** Emerging themes, sub-themes and categories by cadre of health professionals

Facility managers (FMs)	Sub-district/district/provincial/health information managers	M&E and PMTCT program managers
1. Use of information		
• Selective use for reporting	• Selective use of information for monitoring outcomes for political purposes; reporting and campaigns;	• Selective use for reporting • Inadequate use of data for planning purposes
	• Different perspective on data-use	
2. Barriers to information-use		
• Lack of trust in the data		• Data not trusted
• Willingness and attitude of users • Lack of culture of information-use	• Lack of skill to interpret data for planning • Lack of culture of information-use	• Packaging of information • Lack of culture of information-use
• Ripple effect of lack of staff at facility level		• Lack of skills to use data
• Timeliness of feedback on information		• Lack of accountability for accurate data
3. Data quality issues		
• Perceptions of ‘poor-quality data’ • Inaccurate information provided by patient	• Perceptions of ‘poor-quality data’	• Staff at different levels involved in several registers for one program
		• Cooperation between staff at facility level
4. Reasons for poor data quality a) Human resources		
• Staff attitude of non-care	• Staff attitude of non-care	• Three data-collection tools for one program
• Human error in data collection • Work overload/staff turnover • Lack of trained data capturers/clerks	• Clinicians unprepared/lack of skill for data collection • Staff rotation and turnover • Lack of feedback/data interrogation	• Follow up issues: results not captured • Migration of parents/mothers • Access to/tracing all babies born into the cohort
• Lack of ownership of RHIS process • Limitations of supervisory visits/monthly audit	• Lack of numeracy skills	• Lack of feedback/data interrogation • Lack of accountability
b) Equipment		
• Lack of resources and equipment for recording		
c) Validation issues		
• Lack of skills for validation	• Lack of validation at facility level, burden on higher levels • Snowball effect of non-validation at facility level • Extent of validating and coordinating overwhelming at district level	• No proper data validation at sub-district level
d) Training		
	• Structure and content of training should be interactive and experiential in nature	• Experiential training/applied learning based on adult learning principles as preferred option which should be relevant, participatory, learner-centered, ownership
		• Staff turnover challenge in training

### Information-use perceptions

There were mixed feelings among participants about information-use for managing programs and service delivery across all levels. Despite concerns about data inaccuracy, district health managers (DHM), which includes district and program managers, confirmed using the data since it gives a sense of what needs to be addressed.

Participants agreed that the information had been selectively used, either for monitoring outcomes, political purposes, reporting to the next level, or for publicity and/or campaign purposes. One provincial manager claimed that data were widely used for measuring program output:

*“I report on about five different platforms, most of the PMTCT data ... lots of the information I use for reporting purposes, many of them measuring output and program performance … and then in terms of business plan and budget there’s some of them where, especially feeding options, whether we need to procure less or more milk …”* ***(DHM #2)***

However, there were concerns about the lack of information-use by senior management to drive programs, improve service delivery, and inform decision-making for developing action plans. Some ascribed their lack of commitment to health-information tasks to the absence of evidence that the information was used to address problems such as lack of resources and patients’ access to care. Some of the managers saw data collection as a waste of time, as the data were not used. For example, one district manager stated that:

*“They use the figures as to say our program is doing very well ... but besides looking at the data what else are they looking [at]…? Do they talk to the data as well?...We have HH and MK hospitals that are in the middle of nowhere. There are no taxis going [in] that direction… What is the government doing about that? Are they providing any transport… for patients?”* ***(DHM #14)***

A different dimension to information-use was highlighted by a district manager, who argued that program and facility managers are often caught up in managing problems without first analyzing the source of the problem.

*“…Most managers are [so] busy managing problems that they don’t have enough dedicated time to sit down and plan with the data. I think there is not enough attention paid to it and often decisions are being made in a way that does not consider the important little aspects required when collecting data and I think it’s a lack of maybe understanding people when it comes to managing a facility.”* ***(DHM#12*** *)*

### Barriers to information-use

Some of the key barriers to data-use, which emerged, included a lack of trust in the quality of data; a lack of understanding and skills to interpret and use data; information packaging; lack of accountability for accurate data; timeliness of feedback on information, and, a poor information-use culture.

#### Lack of trust in the information collected

Almost all the participants highlighted trust as a major reason for non-use of information. A general consensus was that most managers did not bother to use the information because they did not trust the data captured from the registers.

*“… There are many managers out there; they will very proudly tell you that they don’t bother with the information because it is just rubbish and they are very proud of it ... I wouldn’t trust the information at all … When you look at the information with a magnifying glass, you see many holes. … I am very confident that we are deluding ourselves.”* ***(DHM #12)***

Despite lack of trust in the data, some district health managers claimed that working with poor data was better than not using it; and that one of the ways of improving data quality was to use the data. As indicated:

*“… I feel that even when the information is not good, the only way to make it good is to use it. I am not waiting for the information to be good to use it, because it is never going to be good if you don’t use it. So using poor information that is unreliable and undependable, is always the starting point; it is only in the process of analyzing it, making sense of it and giving feedback to the people who produced it, so that they understand what the data is used for, that you really get information to be good.”* ***(DHM #12)***

#### Lack of understanding and skills to interpret and use data

The general perception was that personnel, especially FMs, lacked the skills to understand and interpret data for planning and that information was reported without being interrogated to check if it was usable. Participants emphasized the importance of strengthening FMs’ understanding of the rationale for collecting data, stressing that once they understood the importance of data, more attention would be given to ensure that data signed off and sent out of the facility was of good quality. The following remark demonstrated the issue of lack of skills:

*“… if I do not understand the data myself, I don’t know what the statistics are saying. There is no way I can use it. So I think it’s probably a lack of skills on the part of the CEOs. They don’t know what these figures represent …”* ***(DHM #6)***

The issue of professional nurses’ inability to interpret data, as a result of their lack of numeracy skills, was also highlighted. Participants claimed that this issue had nothing to do with color or race. The problem was even more pronounced because it is mostly professional nurses who function as FMs and are expected to make decisions about patient care and oversee the day-to-day running of the clinics.

*“I think that a lot of people are slightly nervous of figures. There’s a kind of numeracy mental block …”* ***(DHM #11)***

*“People don’t even understand what a percentage is. … I remember giving feedback to nurses, professional nurses. These were not even from Khayelitsha, so it is not like ‘oh the blacks have got bad mathematical skills’ not at all. … I will say 80% and they will say ‘what do you mean 80%? We only saw 19 patients ...’ So they thought I was saying they saw 80 patients.”* ***(DHM #12)***

Narratives also suggest that the lack of skills to understand and interpret data stemmed from a poor information-use culture. Participants also attributed the inability to use information to a lack of training of managers, especially FMs to use data; they therefore just collected and transmitted data to the next level.

*“We give the information to the senior managers. They are able to use the information for planning. People believed that you collect information; you send it to district, to province, national, then national will see what they want to do with that information.”* ***(DHM #6)***

Nonetheless, district and program managers argued that FMs needed to acquire the necessary skills to use information, and to ensure that the data produced are validated and of good quality, before such data are signed off. They indicated that FMs should be held accountable for decisions made based on invalid information.

*“We need to capacitate managers on the ground on how to use that data. And then to ensure that all the managers … actually sign off the data to ensure it is valid, and to hold managers accountable for decisions taken.”* ***(DHM #10)***

#### Packaging of information

The way information was packaged was of vital importance in determining whether it was used or not. Participants argued that most staff at district and facility levels were not numerically inclined, and were not used to figures and numbers, therefore extra effort was required to interpret the information. They suggested that information should be presented by M&E officers and program managers in a simple, easy-to-understand, and usable format.

*“I think that a lot of people are slightly nervous of figures. There’s a kind of numeracy mental block … you have to find a way to package the information, in [a]digestible way, like sugar coat it almost. You know I think a lot of people aren’t used to looking at data … we will be given all the provincial data and then there’s just these Excel spreadsheets and there are numbers everywhere ... but the M&E manager sort of packaged it in a way that she’d show us these graphs … we had someone who could distil and interpret and give it to us, so if anyone asked practically anyone in the program, nurses, counsellors … they could tell you [interpret the data].”* ***(DHM #11)***

#### Lack of accountability for accurate data

Most of the district managers were concerned that staff at the facility level were nonchalant in the way they performed their daily duties, owing to lack of supervision and accountability. They stated that processes to monitor data accuracy should be put in place at all levels, suggesting that all managers be held accountable for ensuring that data were validated on a monthly basis before being signed off.

*“I think managers need to take responsibility and be accountable for their districts and for their sub-districts. And making sure that what they are signing off on a monthly basis is correct …”* ***(DHM #1)***

### Data-quality issues

Data-quality issues relate to participants’ perceptions of the quality of PMTCT data generated at the facility level. This theme was categorized into four sub-sections: perceptions of poor-quality data; staff at different levels involved in several registers for one program; inaccurate information provided by patients; and, cooperation between staff at facilities.

#### The perception of poor-quality data

All study participants were concerned about the quality of data they received from the facility and agreed there were problems with the data-collection processes.

*“A major problem which we actually found was that there were about six discharges in the pediatric ward, and maybe I don’t know whether the lady (nurse) was drowsy or something, she will put that [*sic*]six under deaths; you see now that’s a HUGE problem.”* ***(FM #5)***

#### Incorrect information provided by patients

Participants expressed their concern about the nature of information received from patients, claiming that patients sometimes gave wrong information to nurses during their clinic visits. The issue here was that if the information fed into the system was inaccurate from the source, this would cause a ripple effect right through the system to the national level, and no amount of validation would solve the problem.

*“I don’t know why people lie about where they live ... that is one of the barriers, people losing documents due maybe to a fire or being robbed or misplacing them completely ... we have foreigners, quite a number of Somalis, Ugandans and Kenyans coming in and there is a language barrier at times.”* ***(DHM #5)***

*“... data collection I would say is quite problematic; what we need to focus on is the source where data is initiated, where data moves from two different points … if the data that you put in there is wrong, then even the system will give you wrong information at the end of the day. It goes a very long way.”* ***(FM#7)***

Another issue raised relates to the nature of the recorded data. A district manager expressed concerns that the issues surrounding poor-quality data were multifaceted. The manager argued that if nurses had the skills to check data accuracy, they should have been able to detect wrong information, stressing that even when the information from the client was correct, they sometimes cannot make meaningful sense of the data.

*“I don’t think it is one thing. I think what happens in the facilities contributes ... they [clients] still sometimes bring wrong data, and then that wrong data is captured in the system. But also, correct data is captured and it says other things, and also if wrong data is brought, someone that is capturing should be able to see that this is abnormal.”* ***(DHM #5)***

#### Staff at different levels involved in several registers for one program

Program coordinators and managers of the PMTCT program, on the other hand, highlighted challenges with how the program was managed. They felt that one of the challenges for data quality was that the PMTCT program was managed by different people at different levels, and involved multiple data-collection tools. Data for the PMTCT program were extracted from three registers managed by different staff. At times the registers are simultaneously used by different nurses to record care provided to patients. When a nurse is busy with one register, the others improvise and find other means of recording the information, such as on pieces of paper for onward transmission to the registers. Sometimes the nurses forget to capture this information onto the registers, or sometimes misplace the loose sheets, hence under-reporting the services provided.

*“There are issues at different levels of data collection and reporting ...different people on the ground are responsible for different registers and, in fact, for some registers, you need more than one provider [nurse]. So, for example, the HCT register is done [captured] by four counsellors and they also need the nurses to provide some of the information. And so I think one of the problems is that they don’t actually work in the registers, they have bits of paper and they put the various bits together in the register later on… but there’s room for error obviously if one person’s piece of paper gets [*sic*] missing…”* ***(DHM #11)***

### Reasons for poor-quality data

When asked about their experience with producing data, participants agreed that the quality of data they produced is suboptimal; however, they gave reasons for this. These were categorized into human resources, equipment and validation issues.

#### Human resources: Staff are not skilled and equipped to record data

There was a consensus that lack of manpower and basic capacity/competence for recording and validating data were the most burning issues. In this regard, FMs strongly emphasized the following human resources issues as some of the reasons that may impede data quality: staff attitude; human error; staff turnover; lack of numeracy skills; insufficient feedback and supervision; and, lack of ownership.

#### Staff attitude

Staff attitude toward performing RHIS tasks, was emphasized as a factor hindering the collection of good-quality data. Concerns were raised about the nonchalant attitudes staff displayed when it comes to data collection. Participants claimed that some staff willfully refused to tick off relevant data, and did not bother to fill in the registers, and when they did, they sometimes did not do it properly or comply with the guidelines.

*“... some clinicians either forget or willfully don’t tick off the relevant data ... it could be there is staff attitude, like someone saying ‘it’s not my job kind of thing, it’s someone else’s’.”* ***(DHM#9)***

*“The staff don’t record properly ... They don’t comply with the tally sheet ... And sometimes we don’t get enough time to check thoroughly and then I sign them and the statistics goes [*sic*] to the next level and you find that there are mistakes which were overlooked ... sometimes other registers are not counted by mistake, which means we don’t count everything that we have done for that month.”* ***(FM #8)***

#### Human error in data collection

Human errors such as forgetfulness, absentmindedness, and carelessness related to issues of staff attitude, were identified as reasons for poor data quality at the facility level. FMs claimed nurses often forgot to record the care given and mistakenly recorded wrong values. The views of some of the participants are presented below:

*“I don’t think nurses tick every patient that comes in for care; sometimes they will just dish out the tablets for those who come for their doses and then maybe after four or five patients, they will remember to tick and suddenly they just tick twice. Sometimes they may tick less or tick more, so it’s difficult to keep accurate stats.”* ***(FM #1)***

*“Sometimes they [nurses] forget what to put where, when they do data collection. Then there are also human errors of course, you count 10 people, and then write 11 by mistake …”* ***(FM #5)***

#### Lack of feedback and limited supervision

Supervision and feedback are important issues related to human error and staff attitude discussed above. Participants argued that through feedback, staff were able to identify areas of their outputs that needed improvement. Despite the importance of feedback, it is often not provided to staff at the facility level.

*“I don’t think there’s nearly enough feedback to people on the ground … it’s taken us many months ourselves to sort of work out how the data flow works … I’m seeing more and more the importance of giving feedback … but I don’t think it happened very much at all.”* ***(DHM #11)***

In addition, the frequency and quality of supervisory visits were also highlighted as a reason for poor-quality data. One FM expressed her concerns about the quality of supervision they receive at the facility level. She argued that supervision is not always adequate, and suggested that supervisors should be more involved in their supervisory activities, rather than just checking a few folders that may not reflect the true nature of what is happening at the facility.

*“During the supervisory visits, sometimes the program manager will pull a few folders to look at what we are doing, but it does not give the true reflection because two folders cannot give you the true picture ... because the folders might all be correct.”* ***(FM #1)***

#### Work overload and staff turnover

Further reasons raised by participants for poor data quality are problems relating to work overload and staff turnover. Managers at different levels argued that it is extremely difficult to produce accurate data with too few staff. They highlighted the challenge of inadequate capacity and manpower to carry out tasks in an already overburdened health system.

*“It is difficult to keep accurate statistics, because the same person who is taking the statistics is the same person who has to give the tablets, it’s the same person who has to find the folder, the same person who must listen to the entering patient, you see. So I don’t think it gives the true reflection.”* ***(FM #1)***

Work overload at the facility level relate to issues of staff shortages and staff rotation, which managers believed were major factors influencing the quality of data produced. Managers asserted that the high staff turnover experienced at both the sub-district and facility levels created a demand for training new staff. Staff turnover was also considered to contribute to situations where data are left uncaptured for months because new staff often lacked the skills and experience to perform HIS-related tasks.

*“In certain districts you have quite a high staff turnover which makes it very difficult, and even though we have a lot of people at district level, you can only train every X amount [*sic*] of weeks or months ... Again staff turnover, even at sub-district level with data capturers, there’s often staff turnover; that is why for a month or two the data will be in shambles like now, and then it’s maybe just a new person who had a totally different idea or didn’t understand.”* ***(DHM#2)***

Furthermore, managers argued that because of staff shortages, people not only multi-task, but are asked to perform tasks they are neither qualified nor trained for.

*“… majority of the facilities don’t have information clerks and now you have to rely on the facility managers sometimes to provide the reports and they also have a lot that they need to do and sometimes the report comes late. It’s not always on time and there are gaps and so on.”* ***(DHM#13)***

#### Lack of numeracy skills and skills for recording and analysis

Our study revealed that most clinicians were unprepared and lacked the necessary skills for data collection at the facility level. A major issue highlighted was clinicians’ lack of understanding of the definitions of some data elements and indicators. Participants claimed that some clinicians did not have proper training on the definitions of the PMTCT data elements and how the elements should be recorded as reflected in the PMTCT protocol.

*“The biggest problem is on the definitions ... some clinicians are not clear with the definitions on the routine monthly report data … a lot of the times the clinicians and nurses are just expected to collect data. They haven’t really been inducted into what are the elements, and what are the definitions.”* ***(DHM#8)***

Lack of numeracy skills also emerged as a reason for poor data quality. Despite the importance of numeracy skills in the data-collection and analysis processes, the majority of the staff involved with data collection, such as clinicians, nurses and data capturers, were reported to have inadequate numeracy skills.

*“I even asked the clinic nurses to come and learn arithmetic, you know the percentage, because we have to do percentages. I arranged with Damelin College because people couldn’t count … but they did the maths and they said they were bored … I nearly went crazy. A child was weighing 4.5kg and when one is having diarrhea, we use the formula of 20ml per kg. Sisters couldn’t calculate that; they would calculate with 5kg, make it to the nearest! You may be endangering the child’s life by given him an overdose.”* ***(DHM #7)***

*“And then there’re some of us who are not all that gifted with numeracy skills, but you know, I think I have a pretty good grasp of it. So I think those are the main challenges.”* ***(DHM #10)***

Another challenge highlighted, also related to skills, was that data capturers/support clerks are not adequately trained to perform their tasks. Even when training was provided, staff reportedly still did not get it right.

*“At the SH clinic the problem was that the person in the baby clinic was not even a professional nurse. So it was a staff member who wasn’t adequately qualified anyway to do the job, and he certainly didn’t understand the register. … In the facilities the staff had never had any training on the use of registers, … and there was actually a new baby follow-up register, and they didn’t fully understand how to use it…”* ***(DHM#11)***

*“I give them training on how to record several times but you know because of this shortage of staff sometime people are just rushing things and then sometimes they think that recording is just waste of their time.”* ***(FM#8)***

One district manager pointed out that a major reason why staff did not benefit from training was the caliber of the people trained in HIS tasks. She argued that most of the trainees are not even qualified to participate in the training.

*“… I was so upset when I learnt about people that were actually trained to be data capturers; one was working in the kitchen, the other one was working there as a cleaner, and I was worried that anyone can just be pulled to do data capturing …”* ***(DHM #7)***

#### Lack of skills for validating and analyzing data

Despite the importance of data validation in ensuring good-quality data, the study shows that staff at district and facility levels reportedly lacked the basic skill to validate data. One FM argued that if she did not have the skills to tell if data were correct or not, no one else in the facility would be able to detect problems with the data.

*“I don’t have skills to sit down and say that and be confident that this is what it is ... [there is] nobody with the skill in the facility.”* ***(FM #1)***

*“You know it’s quite embarrassing actually … [it is] confusing to look at that PMTCT document and validate it, especially when there are sometimes confusing figures ... since I cannot tell whether this is correct or not, my data capturers will never know what is right and what is wrong.”* ***(FM #5)***

#### Training for data collection and capturing/validation

Although the majority of the participants regarded training for recording and validation as necessary for improving data quality, the general agreement was that the structure, content and mode of delivery of such training should be based on adult-learning principles, which should be interactive and experiential in nature. They suggested that the preferred option should be in-service training that is relevant, participatory and learner-centered. However, district managers expressed their frustration about staff behavior during training.

*“I’m tired of training. I would prefer on-the-job training. I believe… [it] is more meaningful than a classroom session whereby they are just happy that they are out of their workplaces. Immediate support, even if it could be a central training … at a facility level…, because it is practice that will make you perfect … I do think that it is very important to strengthen people’s understanding of the reason why data is collected.”* ***(DHM #7)***

*“My experience is that no matter how much training they have people don’t really listen; they only hear what they are expecting to hear …”* ***(DHM #12)***

## Discussion

Despite an increasing demand for data, and the investments and efforts made to collect routine facility-based data, the majority of decisions made by health managers, in terms of planning and resource allocation for managing HIV programs, are not based on data. This study has shown a limited use of data at the facility level for decision-making purposes. Even though about 70% of the surveyed facilities have mechanisms and processes in place to promote information-use, only about half have evidence to show that information was adequately used for the day-to-day management of maternal and child health programs. In spite of the availability of reports in almost all the surveyed facilities, only half claimed to have used the information for advocacy purposes; for mobilizing resources; for adjusting personnel; or for reviewing strategies to achieve performance targets.

The qualitative component of this study concurs with the findings from the facility inventory. Managers across the board confirmed that information was selectively used at the facility, district and provincial levels; however, the study revealed that information is not used for decision-making and planning purposes, but for reporting and monitoring of program outputs such as the proportion of HIV-positive pregnant woman who received Nevirapine prophylaxis. These findings support other studies on information-use [13, 23, 25]. For instance, Peersman et al. highlight the extent to which information is used for planning, management, and implementation of HIV programs. The authors noted that more than half of the 120 countries surveyed globally, self-rated their performance in terms of data-use as below average [25].

A major reason highlighted in this study for the suboptimal use of information is the inability of staff at the facility and district levels to analyze, interpret and use data. This contradicts the expectations of the South African National Department of Health, outlined in the current District Health Management Information System (DHMIS) standard operating procedures (SOPs) [26]. Despite the inclusion of these tasks in the SOPs as key responsibilities of facility and program managers, these activities have not been enforced. According to the SOPs, FMs and professional nurses are not only expected to use data, but also to have the skills to analyze and interpret data. The inability of personnel at both the facility and district levels to use information would imply that effective training and support interventions for data-use have not been fully implemented. These results support other studies that highlight the inadequacies of managers to analyze, interpret and use data [32, 36–38]. Burn and Shongwe’s telephone survey of 27 hospitals, highlight the insufficient use of information by hospital managers to manage service delivery at the hospital level [32]. The authors attributed these inadequacies to the managers’ lack of skills to use data. Since most managers lack these skills to analyze and interpret data, senior management, including M&E managers should package information in a format that allows easy interpretation, to encourage increased information-use amongst managers.

The in-depth interviews identified several barriers to information-use for planning and management purposes, which stem from human-related and organizational issues such as the lack of trust in the data (a situation where the data integrity is questionable), and a lack of a culture of information-use at facility and district levels. The general perspective in this study was that most managers did not use information because they did not trust the data. The lack of trust hinges on inaccurate and incomplete reporting influenced by human, organizational and technical factors. These factors include the lack of core competencies for data collection and analysis [13, 31, 38–40]; staff shortages and work overload [39]; staff attitudes towards RHIS tasks; shortages of basic paper-based resources like registers, tally sheets and stationery [13, 40, 41]; and the lack of information-use culture [5, 12–14]. Unlike Burn and Shongwe [32], Ledikwe et al. and Chaulagai et al. considered additional barriers to information-use such as the lack of data ownership which also emerged from this study [14, 42]. These findings, which support the proposals by Nutley and Reynolds [4] to strengthen managers’ capacity to use information, are consistent with studies by Solarsh and Goga [30] and Mate et al. [6], who argue that the lack of reliable data impede the tracking of progress on health-service delivery.

Regardless of the data-trust issues, suggestions have been made to use poor data. Participants argued that working with poor data is one of the ways of improving data quality, since it is only by analyzing and making sense of the data that one is able to provide feedback to data producers on how to improve data quality. This proposal conforms with the data-use approach intervention suggested by Braa et al. [43]. The intervention involves quarterly data-use workshops for health management staff, where routine data are presented and critiqued. This approach has been successfully used in Tanzania [43] and Rwanda [44] to improve data quality, data transmission, data integrity and data-use. Nutley et al., have used the logic-model intervention to improve the use of data in decision-making in Ivory Coast [18]. A data-collection and feedback training intervention has also been used to improve the quality of routinely collected data in South Africa [45].

Another significant finding of this study is the lack of a culture of information-use for decision-making. This study found an insufficient information-use culture at the surveyed facilities and inadequate evidence of information-use by senior management for planning and decision-making. If there is no demand for information from senior management, and staff do not understand the usefulness of information, chances are FMs will not use information. This result supports findings from other studies [1, 4, 12, 46] that report on the culture of information-use. Kamadjeu et al. highlighted leadership issues as one of the challenges faced in the adoption and sustainability of an electronic health care record in Cameroon. They observed a 50% dropout rate among users of the system, attributed to a lack of support of the new technology by the new management staff, which did not promote a culture of use [46]. Lorenzi et al. have also argued that the success or failure of an organization, irrespective of the management techniques, depends on the leadership [47]. Taylor [48] proposed three main factors for effective adoption of culture change: behaviors - of those we admire, or perceive as role models; symbols - such as who gets promoted; and systems - such as rewards, and punishments. Taylor argued that it is fruitless to try to change individuals’ behavior without first changing the culture of the environment in which they live. In other words, if top leadership promotes a culture of information, personnel are likely to emulate them [48, 49].

## Conclusion

This study shows that information is inadequately used by health managers to inform decisions and for planning purposes, but is selectively used for reporting and monitoring program outputs at the provincial level. The inadequate use of information has been attributed to several behavioral and organizational barriers, which include a lack of trust in the data; the inability of many managers to analyze, interpret, and use information, owing to insufficient skills and, the lack of or an inadequate culture of information-use at the district and facility levels.

The inability of facility and program managers to use information implies that decisions for program implementation and improving service delivery are not always based on data. Limited information-use could have a negative impact on service delivery. Hence, facility and program managers should be provided with practice-based, in-service training, that is participatory and learner-centered, and be supported to use information for planning, management and decision-making. Furthermore, a culture of information-use at all levels needs to be promoted by senior management using proven data-use interventions. Capacity in information-use competencies such as data analysis, interpretation, synthesis and presentation, and efforts to improve data quality should be strengthened. Further investigation is needed to determine how decisions for planning and evaluating key programs such as PMTCT are made, and what informs such decisions if not the data.

## Abbreviations

ARV: Antiretroviral
CEO: Chief Executive Officer
DHIS: District Health Information System
DHM: District Health Manager
DHMIS: District Health Management Information System
DIO: District information officer
DM: District manager
FIO: Facility information officer
FM: Facility manager
HAST: HIV/AIDS, STI & TB coordinators
HCT: HIV counselling and testing
HIO: Health information officer
HIV: Human Immunodeficiency Virus
KZN: KwaZulu-Natal
LMICs: Low- and middle-income countries
M&E: Monitoring and evaluation
PHC: Primary Health Care
PIO: Provincial information officer
PMTCT: Prevention of Mother-To-Child Transmission of HIV
RHIS: Routine Health Information System
SDM: Sub-district manager
SOPs: Standard operating procedures
SURMEPI: Stellenbosch University Rural Medical Education Partnership Initiative
UNGASS: United Nations’ General Assembly
WC: Western Cape
